# Intermediate-Range Migration Furnishes a Narrow Margin of Efficiency in the Two-Strategy Competition

**DOI:** 10.1371/journal.pone.0155787

**Published:** 2016-05-24

**Authors:** Yanling Zhang, Qi Su, Changyin Sun

**Affiliations:** 1 School of Automation and Electrical Engineering, University of Science and Technology Beijing, Beijing, China; 2 Center for Systems and Control, State Key Laboratory for Turbulence and Complex Systems, College of Engineering, Peking University, Beijing, China; University of Maribor, SLOVENIA

## Abstract

It is well-known that the effects of spatial selection on the two-strategy competition can be quantified by the structural coefficient *σ* under weak selection. We here calculate the accurate value of *σ* in group-structured populations of any finite size. In previous similar models, the large population size has been explicitly required for obtaining *σ*, and here we analyze quantitatively how large the population should be. Unlike previous models which have only involved the influences of the longest and the shortest migration rang on *σ*, we consider all migration ranges together. The new phenomena are that an intermediate range maximizes *σ* for medium migration probabilities which are of the tiny minority and the maximum value is slightly larger than those for other ranges. Furthermore, we find the ways that migration or mutation changes *σ* can vary significantly through determining analytically how the high-frequency steady states (distributions of either strategy over all groups) impact the expression of *σ* obtained before. Our findings can be directly used to resolve the dilemma of cooperation and provide a more intuitive understanding of spatial selection.

## Introduction

The world consists of populations of reproductive individuals. In most cases, the reproductive success of individuals is determined by not only their own behaviors but also others’ behaviors. Evolutionary game theory provides a mathematical setting for studying the reproductive competition of such populations. So far, lots of theoretical explorations in evolutionary game dynamics have been performed in well-mixed populations [1–10], where each individual interacts with all other individuals with equal probability. There is also a long-standing tradition of research on structured populations through spatial models [11, 12]. Spatial models most commonly involved are games on graphs [13–26], games in phenotype space [27], and games on sets [28]. In those three types of spatial models, the fitness of individuals is dependent on local interactions with ‘neighbors’. For games on graphs, there is a local updating rule meaning that individuals compete with ‘neighbors’ for reproducing or replacing the offspring. However, games in phenotype space or games on sets assume global updating rule, which means that individuals compete with all others for reproducing the offspring. Compared with well-mixed populations, far less investigations have been obtained analytically in structured populations.

For structured populations (including the well-mixed population), it has been proved that the effect of spatial selection (population structure and updating rule) on the evolutionary competition of two strategies can be quantified by a single parameter *σ* called the structural coefficient under weak section (the difference of individuals’ fitness is very small) [29]. Specifically, for a game between two strategies *A* and *B* described by the payoff matrix $\begin{array}{ll} & \begin{array}{ll} A & B \end{array}\\ \begin{array}{l} A\\ B \end{array} & \left(\begin{array}{ll} a & b\\ c & d \end{array}\right) \end{array}$ (each entry is the payoff of the player in the row when interacting with the player in the column), *A* is more abundant than *B* on average in the stationary distribution under weak section if

$$\begin{array}{c} \sigma a+b>c+\sigma d. \end{array}$$ (1)

Note that *σ* is independent of the payoff values *a*, *b*, *c*, *d*.

For games on graphs, there has not been any unified formula for calculating the structural coefficient *σ*. The concrete values of *σ* for games occurring on some particular graphs (Table 1) have been obtained through the known condition under which natural selection favors one strategy over the other (including the critical cost-to-benefit ratio) [29–36]. The birth-death (BD) and the death-birth process (DB) are widely used in those studies. In each generation of the BD process, an individual is chosen to reproduce with probability proportional to his fitness, and then his offspring replaces one of his neighbors randomly. In each generation of the DB process, an individual is chosen randomly to die, and his neighbors compete for the empty spot proportional to their fitness.

**Table 1:** Structural coefficients for different population structures and updating rules.

Population structures	BD^1,2,3,4,5,6^ WF^6,7^ Moran^8^	DB	Additional assumptions
**1.** Well-mixed: an individual interacts with all others equi-probably	$\frac{N-2}{N}$	$\frac{N-2}{N}$	
**2.** Cycle: each individual is connected to its two neighbors	$\frac{N-2}{N}$	$\frac{3N-8}{N}$	DB: *u* → 0
**3.** Star: one individual occupies the center of the star and the others take up the periphery	$\frac{N^{3}-4N^{2}+8N-8}{N^{3}-2N^{2}+8}$	1	BD: *u* → 0
**4.** Regular graphs of degree *k*: each individual is connected to *k* other individuals	$\frac{N-2}{N}$	$\frac{(k+1)N-4k}{(k-1)N}$	*u* → 0
**5.** Individuals have different neighborhoods for game interaction and for evolutionary updating	1	$\frac{(gh+l)N-4gh}{(gh-l)N}$	Interaction graph, replacement graph and overlap graph are regular with degree *h*, *g*, and *l*
**6.** Games in phenotype space: each individual expresses a phenotype and interacts with only those sharing the same phenotype	$\frac{1+4\nu }{2+4\nu }\left(1+\sqrt{\frac{3+12\nu }{3+4\nu }}\right)$		*μ* = *Nu*, *ν* = *Nv* for BD *μ* = 2*Nu*, *ν* = 2*Nv* for WF large *N*, *μ* → 0
**7.** Games on sets: each individual belongs to *k* of *M* sets,and two individuals interact as many times as the common sets they have	$\frac{1+\nu +\mu }{3+\nu +\mu }\frac{K\left(\nu^{2}+2\nu +\nu \mu \right)+M\left(3+2\nu +\mu \right)}{K\left(\nu^{2}+2\nu +\nu \mu \right)+M\left(1+\mu \right)}$		*μ* = 2*Nu*, *ν* = 2*Nv*, large *N*
**8.** Games on islands: an individual is located in one island and interacts with those in the same island	$\frac{\left(1+2\nu \right)\left(3+2\nu +\sqrt{3(1+2\nu )(3+2\nu )}\right)}{2(1+\nu )(3+2\nu )}$		*μ* = *Nu*, *ν* = *Nv*, large *N*, *μ* → 0

*N*: population size, *u*: strategy mutation probability, *v*: phenotype mutation probability (in **6**), set mutation probability (in **7**), migration probability (in **8**).

For games in phenotype space [27] or games on sets [28], a unified formula [37] has been derived for the structural coefficient *σ* as

$$\begin{array}{c} \sigma =\frac{{\langle I_{AA}N_{B}\rangle }_{0}}{{\langle I_{AB}N_{B}\rangle }_{0}}, \end{array}$$ (2)

where *N*_*B*_ is the number of individuals using *B*, and *I*_*AA*_ (*I*_*AB*_) is the total number of games that individuals using *A* play with individuals using *A* (*B*). It is noteworthy that each game played by two individuals using *A* is counted twice for computing *I*_*AA*_. The sign 〈*X*〉_0_ denotes the quantity which is averaged over all states weighted by the steady-state probabilities under neutral selection (all individuals have the same fitness). The formula also holds for the well-mixed populations (particular cases of games in phenotype space) [1] and games on islands (variants of games in phenotype space) [38].

An alternative approach of calculating the structural coefficient *σ* for games in phenotype space [27], games on sets [28], games on islands [38], and the well-mixed populations [1] is through the known condition under which natural selection favors cooperation over defection. The corresponding values of *σ* are summarized in Table 1. The updating rules used in those studies are the Wright-Fisher (WF) or the Moran process. In the WF process, *N* (population size) individuals compete to reproduce *N* offspring proportional to their fitness, and they all die in the next generation. In the Moran process (the special case of the BD process), one individual is chosen to reproduce an offspring proportional to his fitness, and one individual is chosen to die randomly and equi-probably from the population (including the parent).

In this paper, we focus on the competition of two strategies in the group-structured population which is a variant of games in phenotype space [27] and games on islands [38] and is a special case of games on sets [28] (a group in our model can be understood as a phenotype, an island, or a set). In the previous studies [27, 28, 38, 39], the structural coefficient *σ* can only be obtained for large populations. Here, we will give the accurate values of *σ* for any finite population based on the above-mentioned unified formula of *σ*. Moreover, the value of *σ* calculated by us holds for any ‘isotropic’ migration pattern, migration probability, mutation probability, and group number. Although the large population size is explicitly required in those prior studies [27, 28, 38, 39], it is still unknown how large the population should be. To answer the question, the approximate value of *σ* [38] will be compared with the accurate one obtained by us under the same assumptions. The prior study [38] has studied ‘global migration’ and ‘local migration’ which mean that individuals can disperse from any one to any other group and between only the nearest neighboring groups, respectively. Here, we will first calculate the accurate value of *σ* for any ‘isotropic’ migration pattern, and then pay attention to a representative type of migration pattern fully captured by the migration range to better clarify how the migration range influences the evolution. The above-mentioned ‘global migration’ and ‘local migration’ are the special cases when the migration range is the longest one and the shortest one. Therefore, there will be a more comprehensive analysis about the migration range in this paper compared with the prior study [38]. The unified formula of the structural coefficient *σ* [37] shows that it can be calculated analytically through the probabilities assigned to the event that three or two individuals have the given strategies and locations under neutral selection. Our approach for calculating such probabilities is distinct from those in the prior studies [27, 28, 38, 39], and follows the way that the same probabilities are obtained when the optional strategies are more than two (excluding two) in a recent research [40]. In fact, our approach can be expanded beyond the realm of evolutionary game theory, and can be used to make error estimation in many control problems [41–44] and study the effects of spatial diffusion on herbivore outbreak [45–47].

The structural coefficient *σ* is independent of the payoff values, and it can be applied to the concrete game to resolve the evolution of cooperation. The most widely studied games are the prisoner’s dilemma and the snowdrift game which describe the competition of cooperation and defection with the corresponding two-parameter payoff matrices [*b*−*c*,−*c*;*b*, 0] and [*b*−*c*/2,*b*−*c*;*b*, 0] respectively. From Eq (1), natural selection favors cooperation over defection if $c/b<{\left(c/b\right)}^{*}=\frac{\sigma -1}{\sigma +1}$ for the prisoner’s dilemma or if $c/b<{\left(c/b\right)}^{*}=\frac{2\sigma }{\sigma +2}$ for the snowdrift game. The larger the critical cost-to-benefit ratio (*c*/*b*)^*^ is, the more the evolution of cooperation is favored (cooperation is more abundant in the stationary distribution for more values of *c* given *b*). Larger *σ* leads to higher values of (*c*/*b*)^*^, and thus provides more opportunities for the evolution of cooperation. The value of *σ* obtained by us will be used to analyse what conditions including the migration probability, the migration range, and the mutation probability give rise to a larger value of *σ*. We will explore the way that those parameters change the value of *σ* by investigating analytically how the high-frequency (with high steady-state probability) steady state (including the distribution of either strategy over all groups) influences the unified expression of *σ* [37].

## Models and Method

Consider a structured population of size *N* where individuals are distributed over *M* groups arranged in a regular circle and labeled by 1,⋯,*M* in clockwise direction. Individual *i* plays games with all other individuals of the same group and receives the total payoff *p*_*i*_. The fitness of individual *i* is defined as 1 + *wp*_*i*_, where *w* is the selection intensity. The extreme case *w* → 0 is called weak selection and is our focus. The update follows the frequency-dependent Moran process as follows. In each generation, one individual is picked proportional to fitness to be imitated (reproduce), and one individual (including the parent) is chosen randomly and equi-probably to change strategy (die). The newborn offspring adopts the strategy of the parent with probability 1−*u*, otherwise he mutates to one strategy equi-probably. Meanwhile, the newborn offspring stays in the same group as his parent with probability 1−*v*, otherwise he migrates to a new group according to the prespecified migration pattern.

To understand the prespecified migration pattern better, we show it by a one-dimensional lattice consisting of integer points in [1, *M*] and satisfying the periodic boundary condition *j* + *lM* = *j*, where *l* is an integer. Each integer point represents the label of one group. An edge exists between two points if and only if there is a potential single-step migration path between them. In other words, the offspring can migrate to one of the points connected to the point in which the parent resides. We concentrate on the lattices which look the same from every point. Migration patterns of such sort have been called “isotropic” in population genetics [48].

In our model, the strategy of individual *i* is denoted by *s*_*i*_ (1 for *A* and 0 for *B*), and his location is indicated by an *M*-dimensional vector *h*_*i*_ whose *k*_*th*_ entry is 1 if he is in the *k*_*th*_ group and is 0 otherwise. The numerator and the denominator in Eq (2) are given by

$$\begin{array}{c} \begin{array}{c} {\langle I_{AA}N_{B}\rangle }_{0}={\langle \sum_{l,i,j}h_{i}\cdot h_{j}\left(1-s_{l}\right)s_{i}s_{j}\rangle }_{0}-{\langle \sum_{l,i}\left(1-s_{l}\right)s_{i}\rangle }_{0},\\ {\langle I_{AB}N_{B}\rangle }_{0}={\langle \sum_{l,i,j}h_{i}\cdot h_{j}\left(1-s_{l}\right)s_{i}\left(1-s_{j}\right)\rangle }_{0}. \end{array} \end{array}$$ (3)

Each term in the right side can be expressed by the probabilities assigned to the events that three randomly chosen individuals have the given strategies and locations under neutral selection (see S1 Text for the detailed calculations),

$$\begin{array}{c} \begin{array}{c} {\langle \sum_{l,i,j}h_{i}\cdot h_{j}\left(1-s_{l}\right)s_{i}s_{j}\rangle }_{0}-{\langle \sum_{l,i}\left(1-s_{l}\right)s_{i}\rangle }_{0}=N\left(N-1\right)\left(N-2\right)Pr(s_{1}=0,\\ \;\;s_{2}=1,s_{3}=1,h_{2}\cdot h_{3}=1),\\ {\langle \sum_{l,\;i,\;j}h_{i}\;\cdot \;h_{j}\;\left(1\;-\;s_{l}\right)\;s_{i}\;\left(1\;-\;s_{j}\right)\rangle }_{0}=N^{2}\left(N\;-\;1\right)Pr\;\left(s_{1}=0,\;s_{2}=1,\;s_{3}=0,\;h_{2}\cdot h_{3}=1\right), \end{array} \end{array}$$ (4)

where *Pr*(*s*_1_ = *δ*_1_, *s*_2_ = *δ*_2_,*s*_3_ = *δ*_3_, *h*_2_⋅*h*_3_ = 1) is the probability that three randomly chosen individuals labeled 1, 2, 3 satisfy *s*_1_ = *δ*_1_, *s*_2_ = *δ*_2_, *s*_3_ = *δ*_3_, *h*_2_⋅*h*_3_ = 1.

When the optional strategies for an individual are {1, 2, ⋯, *S*}, the general expression of *Pr*(*s*_1_ = *δ*_1_, *s*_2_ = *δ*_2_, *s*_3_ = *δ*_3_, *h*_2_⋅ *h*_3_ = 1) [40] has been given by

$$\begin{array}{c} \begin{array}{c} Pr\left(s_{1}=\delta_{1},s_{2}=\delta_{2},s_{3}=\delta_{3},h_{2}\cdot h_{3}=1\right)=\frac{1}{3MS^{3}}\sum_{z_{1}=M,z_{2}+z_{3}=Mor2M}\sum_{w_{1}+}\\ {}_{w_{2}+w_{3}=S,2Sor3S}\{\sum_{x_{1}=z_{1}+z_{2},x_{2}=z_{3},y_{1}=w_{1}+w_{2},y_{2}=w_{3}}+\sum_{x_{1}=z_{1}+z_{3},x_{2}=z_{2},y_{1}=w_{1}+w_{3},}\\ {}_{y_{2}=w_{2}}+\sum_{x_{1}=z_{2}+z_{3},x_{2}=z_{1},y_{1}=w_{2}+w_{3},y_{2}=w_{1}}\}\Phi (f\left(z_{1}\right),f\left(z_{2}\right),f\left(z_{3}\right),g\left(w_{1}\right),g\left(w_{2}\right),\\ g\left(w_{3}\right))\Psi \left(f\left(x_{1}\right),f\left(x_{2}\right),g\left(y_{1}\right),g\left(y_{2}\right)\right)\exp\left(-\frac{2\pi i}{S}\left(w_{1}\cdot \delta_{1}+w_{2}\cdot \delta_{2}+w_{3}\cdot \delta_{3}\right)\right), \end{array} \end{array}$$ (5)

where

$$\begin{array}{c} \begin{array}{c} \Psi (f\left(x_{1}\right),f\left(x_{2}\right),g\left(y_{1}\right),g\left(y_{2}\right))=\\ \frac{\left(1-u\right)\left(1-v\right)+\left(1-u\right)v\sum_{i=1}^{2}f\left(x_{i}\right)/2+\left(1-v\right)u\sum_{i=1}^{2}g\left(y_{i}\right)/2+uv\sum_{i=1}^{2}f\left(x_{i}\right)g\left(y_{i}\right)/2}{1\;+\left(N\;-1\right)\left(1-u\right)v\left(1\;-\sum_{i=1}^{2}\frac{f(x_{i})}{2}\right)\;+\left(N\;-1\right)\left(1-v\right)u\left(1\;-\sum_{i=1}^{2}\frac{g(y_{i})}{2}\right)\;+\left(N\;-1\right)uv\left(1\;-\sum_{i=1}^{2}\frac{f\left(x_{i}\right)g\left(y_{i}\right)}{2}\right)}, \end{array} \end{array}$$ (6)

Φ(f(z1),f(z2),f(z3),g(w1),g(w2),g(w3))=2(1-u)(1-v)+(1-u)v(f(z1)+f(z2))+(1-v)u(g(w1)+g(w2))+uv(f(z1)g(w1)+f(z2)g(w2))2+(N-2)(1-u)v(1-∑i=13f(zi)3)+(N-2)(1-v)u(1-∑i=13g(wi)3)+(N-2)uv(1-∑i=13f(zi)g(wi)3),ifx1=z1+z2,x2=z3,y1=w1+w2,y2=w3;2(1-u)(1-v)+(1-u)v(f(z1)+f(z3))+(1-v)u(g(w1)+g(w3))+uv(f(z1)g(w1)+f(z3)g(w3))2+(N-2)(1-u)v(1-∑i=13f(zi)3)+(N-2)(1-v)u(1-∑i=13g(wi)3)+(N-2)uv(1-∑i=13f(zi)g(wi)3),ifx1=z1+z3,x2=z2,y1=w1+w3,y2=w2;2(1-u)(1-v)+(1-u)v(f(z2)+f(z3))+(1-v)u(g(w2)+g(w3))+uv(f(z2)g(w2)+f(z3)g(w3))2+(N-2)(1-u)v(1-∑i=13f(zi)3)+(N-2)(1-v)u(1-∑i=13g(wi)3)+(N-2)uv(1-∑i=13f(zi)g(wi)3),ifx1=z2+z3,x2=z1,y1=w2+w3,y2=w1. (7)

*g*(*x*) (*f*(*x*)) corresponds to the structure function of the random walk describing the mutation process (the migration process) along a lineage and satisfies *g*(*S*) = 1 and *g*(*x*) = *g*(*S*−*x*) (*f*(*M*) = 1 and *f*(*x*) = *f*(*M*−*x*)). It is noteworthy that *w*_1_, *w*_2_, *w*_3_, *y*_1_, *y*_2_ (*z*_1_, *z*_2_, *z*_3_, *x*_1_, *x*_2_) can take on only integers between 1 and *S* (*M*) including the boundaries.

For the mutation pattern of our model (*S* = 2, $g\left(x\right)=\frac{1}{2}+\frac{1}{2}\cos\left(\pi x\right)$), *Pr*(*s*_1_ = *δ*_1_, *s*_2_ = *δ*_2_, *s*_3_ = *δ*_3_, *h*_2_⋅*h*_3_ = 1) is given by (see S2 Text for the detailed calculations)

Pr(s1=δ1,s2=δ2,s3=δ3,h2·h3=1)=124M∑x=1M3Ψ1(f(x))+3Ψ2(f(x))+2(Φ1(f(x))Ψ2(f(x))+Φ2(f(x))Ψ1(f(x))+Φ3(f(x))α1),ifδ1=δ2=δ3;3Ψ1(f(x))-3Ψ2(f(x)),ifδ1=δ2≠δ3orδ1=δ3≠δ2;3Ψ1(f(x))+3Ψ2(f(x))-2(Φ1(f(x))Ψ2(f(x))+Φ2(f(x))Ψ1(f(x))+Φ3(f(x))α1),ifδ2=δ3≠δ1; (8)

where

$$\begin{array}{ll} \Psi_{1}(f)=\frac{1-v+vf}{1+(N-1)v(1-f)}, & \Psi_{2}(f)=\frac{\left(1-u)(1-v+vf\right)}{1+(N-1)u+(N-1)(1-u)v(1-f)},\\ \Phi_{1}(f)=\frac{2-u-v+vf}{2+\frac{2(N-2)u}{3}+\frac{(N-2)(2-u)v}{3}(1-f)}, & \Phi_{2}(f)=\frac{\left(1-u)(2-v+vf\right)}{2+\frac{2(N-2)u}{3}+\frac{(N-2)(2-u)v}{3}(1-f)},\\ \Phi_{3}(f)=\frac{\left(2-u)(1-v+vf\right)}{2+\frac{2(N-2)u}{3}+\frac{(N-2)(2-u)v}{3}(1-f)}, & \alpha_{1}=\frac{1-u}{1+(N-1)u}. \end{array}$$

## Results

### The concrete expression of the structural coefficient

The structural coefficient can be obtained by substituting Eqs (8), (4) and (3) into Eq (2),

$$\begin{array}{c} \sigma =\frac{\left(N-2\right)\sum_{x=1}^{M}\left(3\Psi_{1}+3\Psi_{2}-2\left(\Phi_{1}\Psi_{2}+\Phi_{2}\Psi_{1}+\Phi_{3}\alpha_{1}\right)\right)}{N\sum_{x=1}^{M}\left(3\Psi_{1}-3\Psi_{2}\right)}, \end{array}$$ (9)

where (*f*(*x*)) is omitted from Ψ_*j*_ and Φ_*j*_. It is noteworthy that self-interaction is excluded from the calculation of *I*_*AA*_ above. If self-interaction is included, the structural coefficient is

$$\begin{array}{c} \begin{array}{c} \sigma =\frac{{\langle \sum_{l,i,j}h_{i}\cdot h_{j}\left(1-s_{l}\right)s_{i}s_{j}\rangle }_{0}}{{\langle \sum_{l,i,j}h_{i}\cdot h_{j}\left(1-s_{l}\right)s_{i}\left(1-s_{j}\right)\rangle }_{0}}\\ =\frac{N\left(N-1\right)\left(N-2\right)Pr\left(s_{1}=0,s_{2}=1,s_{3}=1,h_{2}\cdot h_{3}=1\right)+N\left(N-1\right)Pr\left(s_{1}=1,s_{2}=0\right)}{N^{2}\left(N-1\right)Pr\left(s_{1}=0,s_{2}=1,s_{3}=0,h_{2}\cdot h_{3}=1\right)}\\ =\frac{\left(N-2\right)\sum_{x=1}^{M}\left(3\Psi_{1}+3\Psi_{2}-2\left(\Phi_{1}\Psi_{2}+\Phi_{2}\Psi_{1}+\Phi_{3}\alpha_{1}\right)\right)+6M\left(1-\alpha_{1}\right)}{N\sum_{x=1}^{M}\left(3\Psi_{1}-3\Psi_{2}\right)}. \end{array} \end{array}$$ (10)

The first equal sign is self-explanation, the second is acquired from Eq (4) and 〈∑_*l*,*i*_(1−*s*_*l*_)*s*_*i*_〉_0_ = *N*(*N*−1)*Pr*(*s*_1_ = 1,*s*_2_ = 0), and the third from Eq (8) and *Pr*(*s*_1_ = 1, *s*_2_ = 0) = 1 − *α*_1_ (see S3 Text for the calculation of *Pr*(*s*_1_ = 1,*s*_2_ = 0)). The concrete expressions of *σ* in Eqs (9) and (10) have no limitations on the migration probability *v*, the “isotropic” migration pattern *f*(*x*), the mutation probability *u*, the population size *N*, and the group number *M*.

### The comparison of the accurate structural coefficient and the approximate one

The large population size has been explicitly required for obtaining the value of the structural coefficient *σ* in the prior studies [27, 28, 38, 39]. To quantitatively determine how large the population should be, the approximate value of *σ* [38] will be compared with the accurate one calculated by us.

For global migration (a migratory offspring equi-probably disperses to any location except his parent’s location), the approximate structural coefficient [38] has been given by

$$\begin{array}{c} \begin{array}{c} \sigma_{ap}=1+\frac{2(3-6M+3M^{2}+\mu -2M\mu +M^{2}\mu )}{\left(3+M+\mu +M\mu )(-3+3M-\mu +M\mu +M\nu \right)}\\ +\frac{2(1-3M+3M^{2}-M^{3}+\mu -3M\mu +3M^{2}\mu -M^{3}\mu +M\nu -2M^{2}\nu +M^{3}\nu +M\mu \nu -2M^{2}\mu \nu +M^{3}\mu \nu )}{\left(3+M+\mu +M\mu \right)\left(1-2M+M^{2}+\mu -2M\mu +M^{2}\mu -2\nu +2M\nu -\mu \nu +M\mu \nu +M\nu^{2}\right)}, \end{array} \end{array}$$ (11)

where *μ* = *Nu* and *ν* = *Nv*. Self-interaction is considered in the above calculation. Under the same assumptions, the structure function of the random walk describing the migration process is

$$\begin{array}{c} \begin{array}{c} f\left(x\right)=\frac{1}{M-1}\left(\cos\frac{2\pi x}{M}+\cdots +\cos\frac{2\pi (M-1)x}{M}\right). \end{array} \end{array}$$ (12)

Obviously, *f*(*x*) = 1 for *x* = *M* and $f\left(x\right)=-\frac{1}{M-1}$ for *x* ≠ *M*. Substituting Eq (12) into Eq (10) and using $2\frac{2-u}{2+\frac{2(N-2)u}{3}}\alpha_{1}+\frac{1-u}{1+\frac{(N-2)u}{3}}=3\alpha_{1}$, we have the accurate structural coefficient as

$$\begin{array}{ccc} & & \begin{array}{c} \sigma_{ac}=\frac{\left(N-2\right)\left(3\left(1-\alpha_{1}\right)+\left(M-1\right)\left(3\alpha_{2}+3\alpha_{3}-2\tau_{1}\alpha_{1}-2\tau_{2}\alpha_{2}-2\tau_{3}\alpha_{3}\right)\right)+6M\left(1-\alpha_{1}\right)}{3N(1-\alpha_{1}+\left(M-1\right)\left(\alpha_{2}-\alpha_{3}\right))}, \end{array} \end{array}$$ (13)

where

$$\begin{array}{ll} \alpha_{1}=\frac{1-u}{1+(N-1)u}, & \alpha_{2}=\frac{1-Mv/(M-1)}{1+(N-1)Mv/(M-1)},\\ \alpha_{3}=\frac{\left(1-u)(1-Mv/(M-1)\right)}{1+(N-1)u+(N-1)(1-u)Mv/(M-1)}, & \tau_{1}=\frac{\left(2-u)(1-Mv/(M-1)\right)}{2+\frac{2(N-2)u}{3}+\frac{(N-2)(2-u)Mv}{3(M-1)}},\\ \tau_{2}=\frac{\left(1-u)(2-Mv/(M-1)\right)}{2+\frac{2(N-2)u}{3}+\frac{(N-2)(2-u)Mv}{3(M-1)}}, & \tau_{3}=\frac{\left(2-u-Mv/(M-1)\right)}{2+\frac{2(N-2)u}{3}+\frac{(N-2)(2-u)Mv}{3(M-1)}}. \end{array}$$

**Fig 1:**
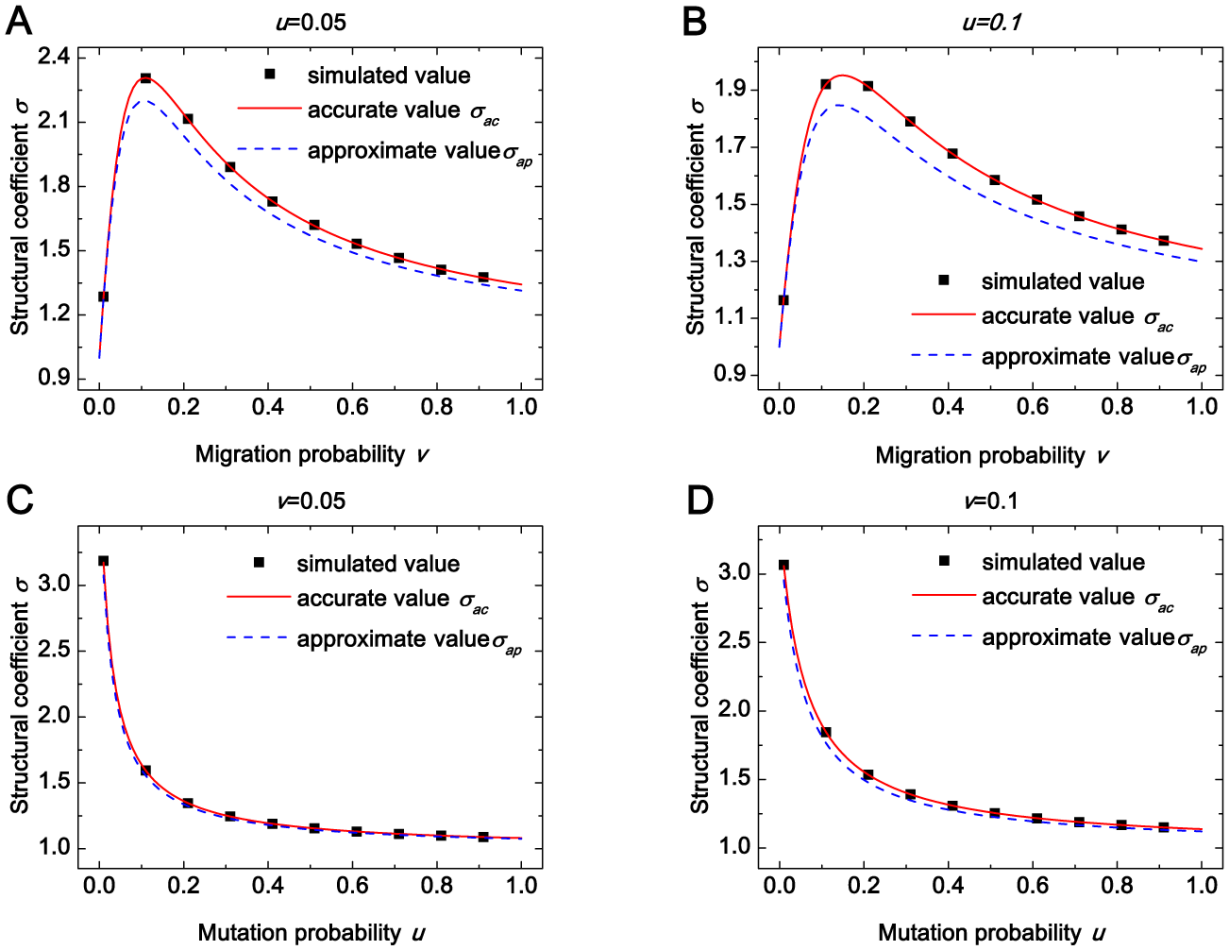
The comparison of the accurate structural coefficient*σ*_*ac*_, the approximate one *σ*_*ap*_, and the one from the Monte Carlo simulation across various mutation probabilities (*u*) and various migration probabilities (*v*). (A)-(D) shows that *σ*_*ac*_ (solid line) is in agreement with the one from Monte Carlo simulations (square, averaged over 10^9^ generations) for all *v* and all *u*, and *σ*_*ap*_ (dashed line) is in line with the one from the Monte Carlo simulation (square, averaged over 10^9^ generations) for low *v* and has a significance difference from the one from the Monte Carlo simulation (square, averaged over 10^9^ generations) for high *v*. Parameters: *N* = 100, *M* = 19.

Fig 1 tells us that *σ*_*ac*_ is in excellent agreement with the one from Monte Carlo simulations for all migration probabilities and all mutation probabilities and *σ*_*ap*_ is in line with the structural coefficient from Monte Carlo simulations only for low migration probabilities. This verifies the accuracy of *σ*_*ac*_ and illustrates the inaccuracy of *σ*_*ap*_ under high migration probabilities.

**Fig 2:**
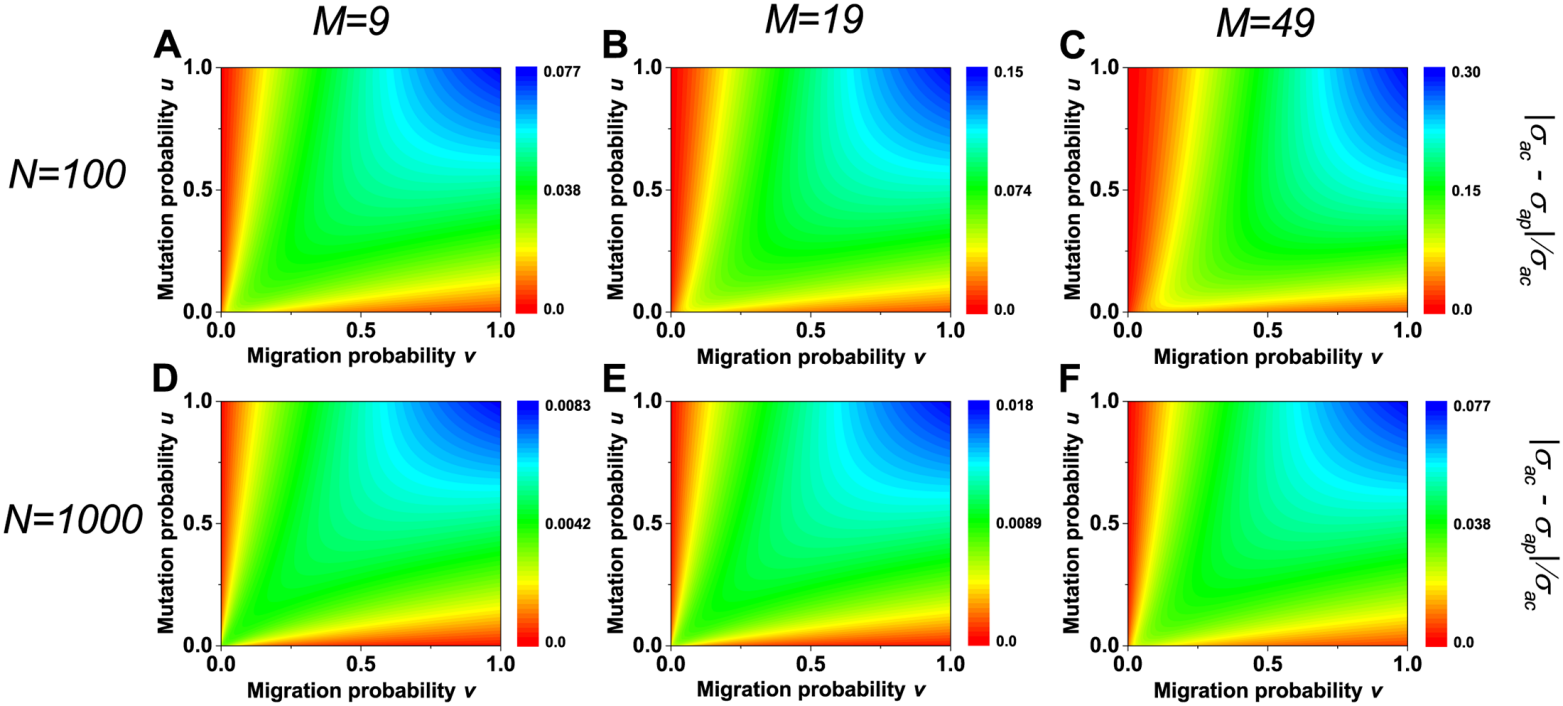
Comparison of the accurate structural coefficient*σ*_*ac*_ and the approximate one *σ*_*ap*_ across the parameter space (*v*, *u*). A population of size *N* = 100 (*A*, *B*, *C*) or *N* = 1000 (*D*, *E*, *F*) is distributed over *M* = 9 (*A*, *D*), *M* = 19 (*B*, *E*), *M* = 49 (*C*, *F*) groups, respectively. The relative difference of *σ*_*ac*_ and *σ*_*ap*_, |*σ*_*ac*_−*σ*_*ap*_|/*σ*_*ac*_, decreases as *N* rises (each column) or as *M* diminishes (each row), and it is small when *u* or *v* is low (each panel). Note the same color in all panels represents different values.

To fully determine the conditions under which *σ*_*ap*_ gives a perfect approximation of *σ*_*ac*_, they are compared for different population sizes (*N*), different group numbers (*M*), all non-zero mutation probabilities (*u*), and all migration probabilities (*v*) in Fig 2. The population size *N* plays a vital role in the comparison of *σ*_*ac*_ and *σ*_*ap*_. Through each column of Fig 2, *σ*_*ap*_ provides a better approximation of *σ*_*ac*_ in large populations (e.g., *N* = 1000) than in small populations (e.g., *N* = 100). The significant influence of *N* on the comparison of *σ*_*ac*_ and *σ*_*ap*_ is resulted from the fact that large populations are required in calculating *σ*_*ap*_ when the coalescence time is approximated to be exponentially distributed. Given *N*, less groups lead to a smaller relative difference of *σ*_*ap*_ and *σ*_*ac*_ as shown in each row of Fig 2. This is because the group number *M* can greatly expand the relative difference resulting from *N*. Given *N*, *σ*_*ap*_ has a smaller difference (red region in each panel) when *u* or *v* is low. The major reason is that the calculation of *σ*_*ap*_ uses the Poisson distribution to approximately describe both the number of mutation events and the one of migration events along a lineage. Accordingly, it has a close dependence on the group number, the migration probability, and the mutation probability how large populations are appropriate for the prior studies [27, 28, 38, 39].

### Effects of migration and mutation on the structural coefficient

**Fig 3:**
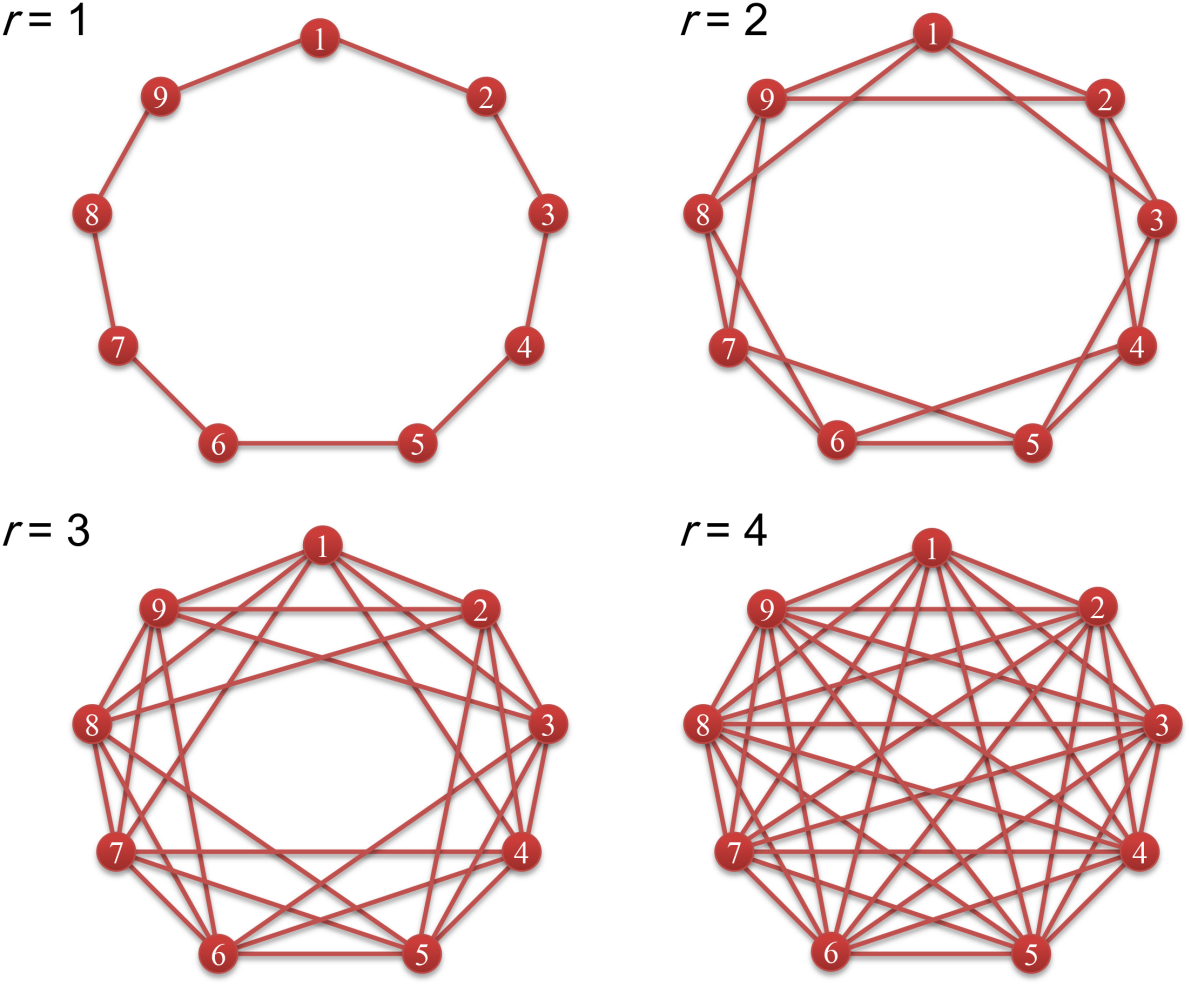
Migration patterns characterized by the migration range*r*. Nine groups (red node) are arranged in a regular circle and labelled from 1 to 9 in clockwise. An edge exists between two nodes if and only if there is a potential single-step migration path between them. In other words, an offspring can migrate to one of the nodes connected to the node in which the parent is located. The distance between two groups takes on one of the values 1, 2, 3, 4. The migration range *r* means that the set of the displacements that a single-step migration leads to is Ω(*r*) = {1,⋯,*r*}.

In our model, *M* groups are arranged in a circle, and thus the distance between two groups is taken from the values 1, 2, ⋯, ⌊*M*/2⌋ where ⌊*x*⌋ is the greatest integer not greater than *x*. To better clarify how the migration range affects the structural coefficient *σ*, we focus on a representative type of migration patterns characterized by the migration range *r* (the largest displacement that a single-step migration leads to). Fig 3 illustrates such migration patterns. All possible displacements that a single-step migration leads to are assumed to form the set Ω(*r*) = {1, 2, ⋯, *r*}, and all elements of Ω(*r*) are assumed to be performed equiprobably. The corresponding *f*(*x*) is

$$\begin{array}{c} \begin{array}{cc} f\left(x;r\right)=\frac{1}{M-1}\left(\cos\frac{2\pi x}{M}+\cdots +\cos\frac{2\pi (M-1)x}{M}\right), & \text{if}\;r=\frac{M}{2}\;\text{for}\;\text{even}\;M\text{;}\\ f\left(x;r\right)=\frac{1}{r}\left(\cos\frac{2\pi x}{M}+\cdots +\cos\frac{2\pi rx}{M}\right), & \text{otherwise.} \end{array} \end{array}$$ (14)

It is noteworthy that the reason why the term sin(⋯) disappears in *f*(*x*; *r*) is the symmetry of migration to the left and to the right direction. Substituting *f*(*x*; *r*) into Eq (9) leads to the value of *σ* for the migration range *r*. It will be used to analyse what conditions including the migration probability, the migration range, and the mutation probability yield a larger value of *σ*. We will show the way that those parameters change the value of *σ* by determining analytically how the high-frequency steady state influences the unified expression of *σ* [37].

**Fig 4:**
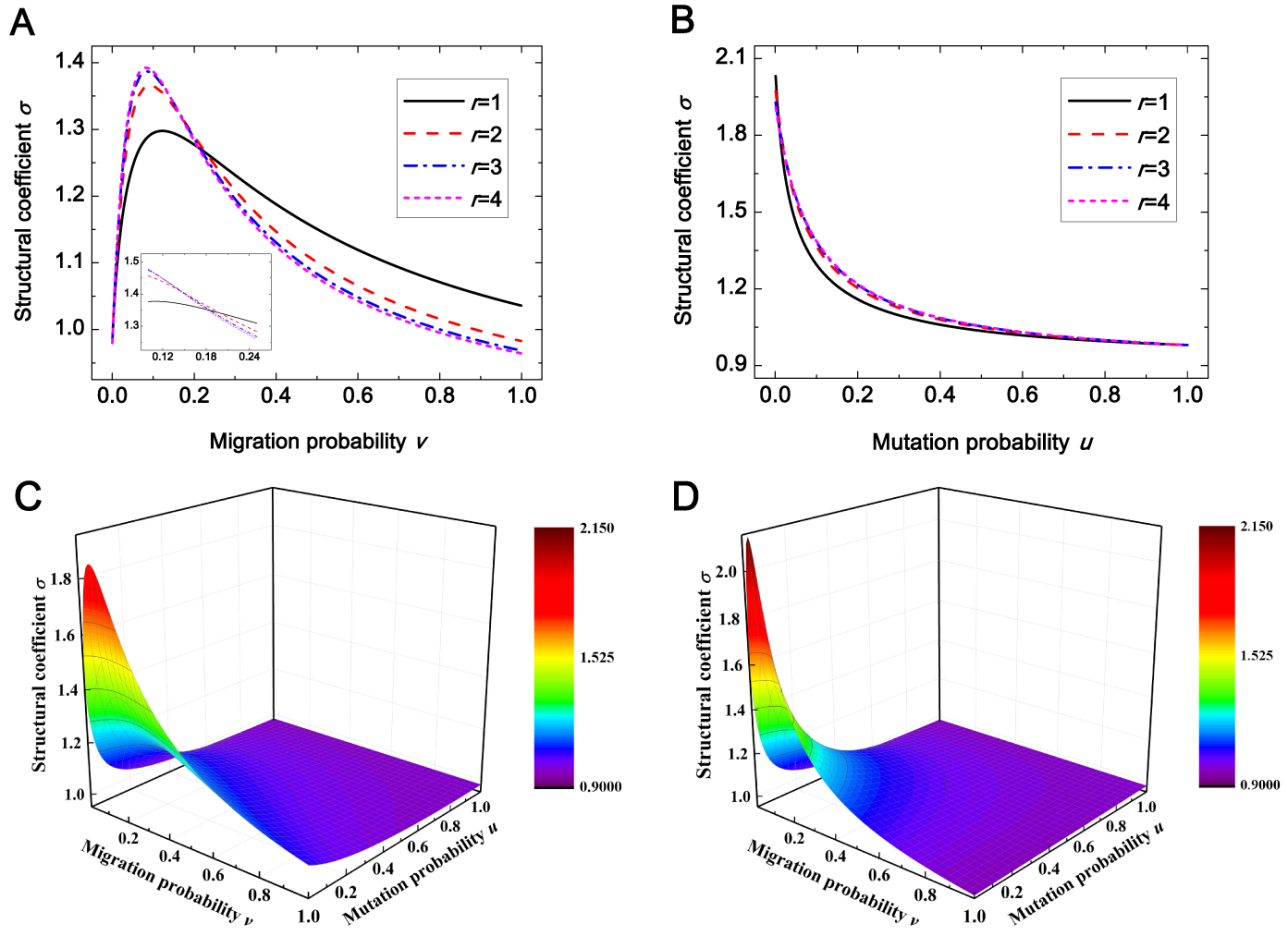
The structural coefficient *σ* depends on the migration probability *v*, the mutation probability *u*, and the migration range *r*. Panel (A) shows that for each *r*, *σ* first increases and then decreases when *v* grows, and among all migration ranges the one leading to the largest value of *σ* is *r* = 4 for low *v*, *r* = 2,3 for medium *v* (see the inset), and *r* = 1 for high *v*. Panel (B) demonstrates that for each *r*, *σ* diminishes as *u* increases. We also give a whole view of how *σ* changes with *u* and *v* for *r* = 1 (panel (C)) and for *r* = 4 (panel (D)) which can verify the universality of the phenomena in panel (C) and panel (D). Parameters: *N* = 100, *M* = 9, (A) *u* = 0.07, (B) *v* = 0.1.

**Fig 5:**
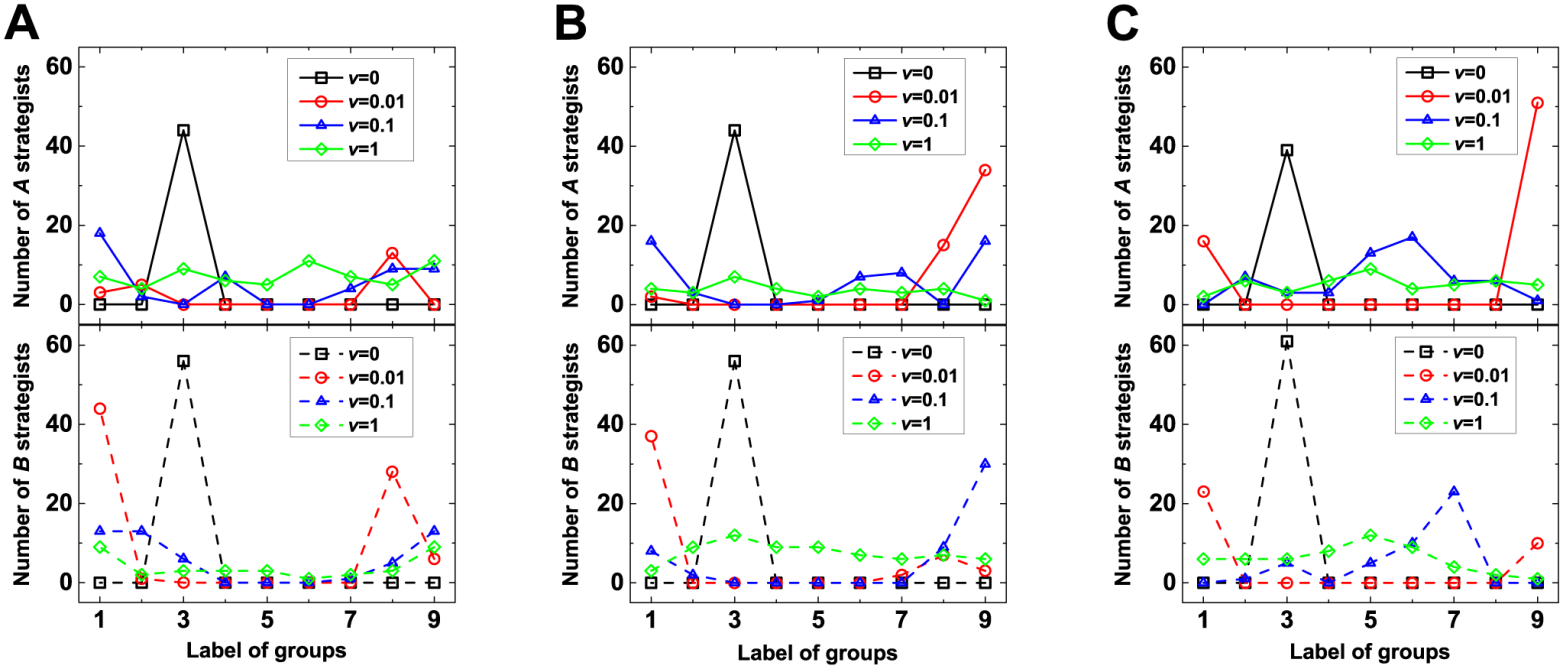
The distribution of strategy *A* and the one of strategy *B* over all groups. The above panels exhibit the distributions of strategy *A* over *M* = 9 groups and the below the distributions of strategy *B* over *M* = 9 groups at generation 10^8^ (A), generation 5 × 10^8^ (B), and generation 9 × 10^8^ (C). For the migration probability *v* = 0 (◻), all individuals are centered in one group; for *v* = 0.01 (◯) and for *v* = 0.1 (△), more groups are taken up and individuals of a given strategy are distributed unevenly over groups; for *v* = 1 (⋄), all groups are occupied and the distribution of each strategy over nine groups becomes more uniform. Parameters: *N* = 100, *M* = 9, *u* = 0.07, *r* = 1.

As shown in Fig 4A, the structural coefficient *σ* is below 1 for the vanishing migration probability (*v* = 0). Here, the state that all individuals are centered in one group is an absorbing state that, once entered, cannot be left (Fig 5). In other words, the long-term population evolves just like the well-mixed population. For the purpose of calculating *I*_*AA*_ *N*_*B*_−*I*_*AB*_ *N*_*B*_ and *I*_*AB*_ *N*_*B*_, the absorbing states can be characterized by a vector (*x*_*A*_, *x*_*B*_) where *x*_*A*_ and *x*_*B*_ are the number of individuals using *A* and the one of individuals using *B*, respectively. Two states (*x*, *y*) and (*y*, *x*) have the same steady-state probability, as there are no fitness differences between *A* and *B* under neutral selection. If they are considered together,

$$\begin{array}{c} \begin{array}{c} I_{AA}N_{B}-I_{AB}N_{B}=-2xy,I_{AB}N_{B}=\left(x+y\right)xy. \end{array} \end{array}$$ (15)

Since *x* and *y* are non-zero in most steady states under neutral selection, 〈*I*_*AA*_ *N*_*B*_ −*I*_*AB*_ *N*_*B*_〉_0_ < 0 and 〈*I*_*AB*_ *N*_*B*_〉_0_ > 0, and thus $\sigma =\frac{{\langle I_{AA}N_{B}\rangle }_{0}}{{\langle I_{AB}N_{B}\rangle }_{0}}<1$. To sum up, the absorbing state that all individuals are centered in one group causes *σ* to be less than one.

As demonstrated in Fig 4A–4D, there exists a medium migration probability that leads to the largest structural coefficient for each migration range. To explain this phenomenon, the characteristics of the high-frequency steady states will be analyzed for different migration probabilities (Fig 5). When the migration probability is non-zero but not high (e.g., *v* = 0.01 or *v* = 0.1), individuals occupy more than one group but not all groups. Meanwhile, individuals using a given strategy are distributed over different groups non-uniformly. However for sufficiently high migration probabilities (e.g., *v* = 1), all groups are full of individuals, and the distribution of each strategy over all groups tends to be uniform.

How the above characteristics of the high-frequency steady states impact the unified expression of the structural coefficient will be illustrated analytically by considering the structure of two groups. In order to calculate 〈*I*_*AA*_ *N*_*B*_−*I*_*AB*_ *N*_*B*_〉_0_ and 〈*I*_*AB*_ *N*_*B*_〉_0_, a state here can be described by

$$\begin{array}{c} \left(\begin{array}{cc} N_{A}^{1} & \;N_{B}^{1}\\ N_{A}^{2} & \;N_{B}^{2} \end{array}\right) \end{array}$$ (16)

where $N_{X}^{i}$ is the number of individuals using strategy *X* in the *i*_*th*_ group. Considering the following four states altogether,

$$\begin{array}{c} \left(\begin{array}{cc} x\;-\;k & y\;-\;m\\ k & m \end{array}\right),\;\;\left(\begin{array}{cc} y\;-\;m & x\;-\;k\\ m & k \end{array}\right),\;\;\left(\begin{array}{cc} y\;-\;k & x\;-\;m\\ k & m \end{array}\right),\;\;\left(\begin{array}{cc} x\;-\;m & y\;-\;k\\ m & k \end{array}\right), \end{array}$$ (17)

we get

$$\begin{array}{c} \begin{array}{cc} I_{AA}N_{B}-I_{AB}N_{B} & =\left(k+m\right){\left(x-y\right)}^{2}+2N{\left(k-m\right)}^{2}-4xy,\\ I_{AB}N_{B} & =N(2xy-k(N-2m)-m(N-2k))\\ & =N(2xy-(k+m)N+4km). \end{array} \end{array}$$ (18)

*k* = *m* = 0 corresponds to the steady states for *v* = 0 or for the well-mixed population. In this case, the values in Eq (18) double the values in Eq (15), as Eq (18) counts two states (*x*, *y*) and (*y*, *x*) twice. By observing Eq (18), the structural coefficient *σ* is enlarged in three ways as follows. (1) When individuals find new groups, meaning *k* ≠ 0 or *m* ≠ 0, *I*_*AA*_ *N*_*B*_− *I*_*AB*_ *N*_*B*_ becomes larger and *I*_*AB*_ *N*_*B*_ smaller than those for *k* = *m* = 0, and hence *σ* increases. (2) When individuals using a given strategy are distributed unevenly over groups, indicating a greater difference between *k* and *m*, both *I*_*AA*_ *N*_*B*_−*I*_*AB*_ *N*_*B*_ and *I*_*AB*_ *N*_*B*_ get larger than a smaller difference between *k* and *m*, and it leads to the rise of *σ* that the former dominates *σ*. (3) When more and more individuals use one strategy than the other, showing the expanding disparity between *x* and *y*, *I*_*AA*_ *N*_*B*_−*I*_*AB*_ *N*_*B*_ gets larger and *I*_*AB*_ *N*_*B*_ gets smaller, and thus *σ* increases.

When the migration probability grows out of zero but is small (e.g., *v* = 0.01, 0.1), individuals take up more than one group (i.e., some find new groups) and individuals using a given strategy are distributed non-uniformly over groups, and thus the structural coefficient *σ* gets larger than the one for *v* = 0. Large migration probabilities (e.g., *v* = 1) mean that individuals occupy nearly all groups and the distribution of a given strategy over all groups tends to be uniform as the migration probability increases, and thus lowers the value of *σ*. Therefore, there exists a medium migration probability leading to the maximum value of *σ*.

Moreover, Fig 4A demonstrates the migration range giving rise to the largest structural coefficient *σ* varies with the migration probability: it is the longest range for low migration probabilities, some intermediate range for medium migration probabilities, and the shortest range for high migration probabilities. This result can be understood as follows. Low migration probabilities prevent individuals from finding new groups. Here, the longest range provides most opportunities for individuals to take up new groups, and thus leads to the maximum value of *σ* among all migration ranges. High migration probabilities raise the likelihood that individuals using a given strategy are distributed relatively uniformly over all groups. In this case, the shortest range prevents the distribution of a given strategy over all groups becoming even to the largest extent, and gives rise to the largest value of *σ* among all migration ranges. The migration probabilities for the above two cases do not cover the entire probability space. For medium migration probabilities, an intermediate range, which balances between the above two cases, maximizes *σ* among all migration ranges. Here, such medium migration probabilities are of the tiny minority and the maximum value of *σ* is slightly larger than the ones of other ranges. Therefore, intermediate-range migration furnishes a narrow margin of efficiency in the two-strategy competition.

From Fig 4B–4D, the structural coefficient *σ* is found to diminish with the increase of mutation probability. This conclusion can be obtained from the expression of $\frac{\sigma -1}{\sigma +1}$ whose monotonicity with respect to the mutation probability is the same as the one of *σ*:

$$\begin{array}{c} \frac{\sigma -1}{\sigma +1}=1-\frac{N\sum_{x=1}^{M}\left(3\Psi_{1}-3\Psi_{2}\right)}{\sum_{x=1}^{M}\left(\left(3N-3\right)\Psi_{1}-3\Psi_{2}-\left(N-2\right)\left(\Phi_{1}\Psi_{2}+\Phi_{2}\Psi_{1}+\Phi_{3}\alpha_{1}\right)\right)}. \end{array}$$ (19)

Obviously, this expression decreases with the mutation probability. In addition, the above conclusion can also be understood through how the high-frequency steady state influences the unified expression of *σ*. In the low mutation probability limit, nearly all individuals adopt the same strategy, and the huge difference between the numbers of individuals using either strategy accounts for the maximum value of *σ*. In the high mutation probability limit, two strategies are present in the population simultaneously with approximately equal number of individuals, and the tiny difference causes the minimum value of *σ*.

## Discussion

For structured populations, it has been proved that the effects of spatial selection (population structure and updating rule) on the two-strategy competition have been quantified by the structural coefficient *σ* under weak selection [29]. The larger value of *σ* provides more opportunities for natural selection to favor the evolution of cooperation in the sense that it yields a larger critical cost-to-benefit ratio. Therefore, the calculation of *σ* is important for investigating analytically the condition for the emergence of cooperation. In this paper, the accurate value of *σ* is calculated in group-structured populations of any finite size. Besides arbitrary population size, the value of *σ* obtained by us is appropriate for arbitrary migration probability, “isotropic” migration pattern, mutation probability, and group number. Our values of *σ* are verified to be in excellent agreement with the results of Monte Carlo simulations. Our model is a variant of games in the phenotype [27] and games on island [38] and is a particular case of games on sets [28]. The values of *σ* in those prior studies [27, 28, 38, 39] can be obtained through the known conditions under which natural condition favors cooperation over defection. In those studies, the large population size has been explicitly required but it is unknown how large the population should be. By comparing the approximate value of *σ* [38] with the accurate one obtained by us, we find that it depends closely on the mutation probability, the migration probability, and the group number how large populations are appropriate for those studies.

The effects of the longest and the shortest migration range on the structural coefficient *σ* have been compared in large populations [38], and it has been found that the longest range leads to the largest value of *σ* for low migration probabilities and the shortest range for high migration probabilities. Unlike the prior study, we consider all migration ranges together in finite populations of any size. Besides the phenomena found in [38], we find a new phenomenon that intermediate-range migration provides a narrow-margin of efficiency for the two-strategy competition. More specifically, migration probabilities for which some intermediate range maximizes *σ* among all ranges are of the tiny minority and the corresponding values of *σ* have small differences among all ranges. Meanwhile, we also study the influence of the migration probability on *σ* and find that a lower mutation probability yields a higher value of *σ*.

Our findings can be directly used to obtain how migration or mutation impacts the evolution of cooperation in any finite population when the value of *σ* is applied to a concrete game, e.g., the prisoner’s game or the snowdrift game. The effects of migration and mutation on the evolution of cooperation have also been implicitly given by a recent research about the two-layered group-structured population [40], as the dynamics of our model (the single-layered group-structured population) is the same as the one of a special case of the two-layered group-structured population (e.g., the fraction of the cooperative level of the first layer in the overall cooperative level is 0 or 1 in that paper). Of course, the value of *σ* for our model can also be obtained through the known cost-to-benefit ratio for the above-mentioned special case. Compared with that study, we not only provide an alternative and more direct approach to calculate *σ*, but also show the ways that migration or mutation enlarges the value of *σ* through determining analytically how the high-frequency (with high steady-state probability) steady state (including the distribution of either strategy over all groups) influences the unified expression of *σ*. Migration enlarges the value of *σ* in the following three ways: (1) individuals find new groups, (2) individuals using a given strategy are distributed unevenly over different groups, (3) more and more individuals use one strategy than the other. However, mutation changes the value of *σ* mainly through the third way. This provides a more intuitive understanding of spatial selection.

## Supporting Information

S1 Text (PDF)

S2 Text (PDF)

S3 Text (PDF)

## References

[1] Nowak MA, Sasaki A, Taylor C, Fudenberg D. Emergence of cooperation and evolutionary stability in finite populations. Nature. 2004; 428(6983):646–650. 10.1038/nature02414 15071593

[2] Traulsen A, Claussen JC, Hauert C. Coevolutionary dynamics: from finite to infinite populations. Physical Review Letters. 2005; 95(23):238701 10.1103/PhysRevLett.95.238701 16384353

[3] Fudenberg D, Nowak MA, Taylor C, Imhof LA. Evolutionary game dynamics in finite populations with strong selection and weak mutation. Theoretical Population Biology. 2006; 70(3):352–363. 10.1016/j.tpb.2006.07.006 16987535 PMC3279757

[4] Zhang Y, Wu T, Chen X, Xie G, Wang L. Mixed strategy under generalized public goods games. Journal of Theoretical Biology. 2013; 334:52–60. 10.1016/j.jtbi.2013.05.011 23702332

[5] Altrock PM, Traulsen A. Deterministic evolutionary game dynamics in finite populations. Physical Review E. 2009; 80(1):011909 10.1103/PhysRevE.80.011909 19658731

[6] Zhang Y, Fu F, Wu T, Xie G, Wang L. A tale of two contribution mechanisms for nonlinear public goods. Scientific Reports. 2013; 3:2021 10.1038/srep02021 23779102 PMC3685828

[7] Taylor C, Fudenberg D, Sasaki A, Nowak MA. Evolutionary game dynamics in finite populations. Bull of Mathematical Biology. 2004; 66(6):1621–1644. 10.1016/j.bulm.2004.03.004 15522348

[8] Imhof LA, Nowak MA. Evolutionary game dynamics in a Wright-Fisher process. Journal of Mathematical Biology. 2006; 52(5):667–681. 10.1007/s00285-005-0369-8 16463183 PMC3279756

[9] Zhang Y, Fu F, Wu T, Xie G, Wang L. Inertia in strategy switching transforms the strategy evolution. Physical Review E. 2011; 84(6):066103 10.1103/PhysRevE.84.066103 22304151

[10] Traulsen A, Pacheco JM, Nowak MA. Pairwise comparison and selection temperature in evolutionary game dynamics. Journal of Theoretical Biology. 2007; 246(3):522–529. 10.1016/j.jtbi.2007.01.002 17292423 PMC2001316

[11] Nowak MA, Tarnita CE, Antal T. Evolutionary dynamics in structured populations. Philosophical Transactions of the Royal Society B. 2010; 365(1537):19–30. 10.1098/rstb.2009.0215 PMC2842709 20008382

[12] Perc M, Gomez-Gardenes J, Szolnoki A, Floria LM, Moreno Y. Evolutionary dynamics of group interactions on structured populations: a review. Journal of the royal society interface. 2013; 10(80):20120997 10.1098/rsif.2012.0997 PMC3565747 23303223

[13] Szabó G, Tőke C. Evolutionary prisoner’s dilemma game on a square lattice. Physical Review E. 1998; 58(1):69–73. 10.1103/PhysRevE.58.69

[14] Szabó G, Hauert C. Phase transitions and volunteering in spatial public goods games. Physical Review Letters. 2002; 89(11):118101 10.1103/PhysRevLett.89.118101 12225171

[15] Santos FC, Pacheco JM. Scale-free networks provide a unifying framework for the emergence of cooperation. Physical Review Letters. 2005; 95(9):098104 10.1103/PhysRevLett.95.098104 16197256

[16] Lieberman E, Hauert C, Nowak MA. Evolutionary dynamics on graphs. Nature. 2005; 433(7023):312–316. 10.1038/nature03204 15662424

[17] Antal T, Redner S, Sood V. Evolutionary dynamics on degree-heterogeneous graphs. Physical Review Letters. 2006; 96(18):188014 10.1103/PhysRevLett.96.188104 PMC2430074 16712402

[18] Ohtsuki H, Nowak MA, Pacheco JM. Breaking the symmetry between interaction and replacement in evolutionary dynamics on graphs. Physical Review Letters. 2007; 98(10):108106 10.1103/PhysRevLett.98.108106 17358573 PMC2387227

[19] Szabó G, Fáth G. Evolutionary games on graphs. Physics Reports. 2007; 446(4–6):97–216.

[20] Helbing D, Yu WJ. The outbreak of cooperation among success-driven individuals under noisy conditions. Proceedings of the National Academy of Sciences of the United States of America. 2009; 106(10):3680–3685. 10.1073/pnas.0811503106 19237576 PMC2646628

[21] Perc M, Szolnoki A. Coevolutionary games-A mini review. Biosystems. 2010; 99(2):109–125. 10.1016/j.biosystems.2009.10.003 19837129

[22] Fu F, Nowak MA, Hauert C. Invasion and expansion of cooperators in lattice populations: Prisoner’s dilemma vs. snowdrift games. Journal of Theoretical Biology. 2010; 266(3):358–366. 10.1016/j.jtbi.2010.06.042 20619271 PMC2927800

[23] Chen XJ, Szolnoki A, Perc M. Risk-driven migration and the collective-risk social dilemma. Physical Review E. 2012; 86(3):036101 10.1103/PhysRevE.86.036101 23030974

[24] Wu T, Fu F, Zhang YL, Wang L. Expectation-driven migration promotes cooperation by group interactions. Physical Review E. 2012; 85(6):066104 10.1103/PhysRevE.85.066104 23005159

[25] Rand DG, Nowak MA, Fowler JH, Christakis NA. Static network structure can stabilize human cooperation. Proceedings of the National Academy of Sciences of the United States of America. 2004; 111(48):17093–17098. 10.1073/pnas.1400406111 PMC4260616 25404308

[26] Perc M, Szolnoki A. Self-organization of punishment in structured populations. New Journal of Physics. 2012; 14:043013 10.1088/1367-2630/14/4/043013

[27] Antal T, Ohtsuki H, Wakeley J, Taylor PD, Nowak MA. Evolution of cooperation by phenotypic similarity. Proceedings of the National Academy of Sciences of the United States of America. 2009; 106(21):8597–8600. 10.1073/pnas.0902528106 19416902 PMC2688992

[28] Tarnita CE, Antal T, Ohtsuki H, Nowak MA. Evolutionary dynamics in set structured populations. Proceedings of the National Academy of Sciences of the United States of America. 2009; 106(21):8601–8604. 10.1073/pnas.0903019106 19433793 PMC2689033

[29] Tarnita CE, Ohtsuki H, Antal T, Fu F, Nowak MA. Strategy selection in structured populations. Journal of Theoretical Biology. 2009; 259(3):570–581. 10.1016/j.jtbi.2009.03.035 19358858 PMC2710410

[30] Ohtsuki H, Nowak MA. Evolutionary games on cycles. Proceedings of the Royal Society B. 2006; 273(1598):2249–2256. 10.1098/rspb.2006.3576 16901846 PMC1635521

[31] Ohtsuki H, Hauert C, Lieberman E, Nowak MA. A simple rule for the evolution of cooperation on graphs and social networks. Nature. 2006; 441(7092):502–505. 10.1038/nature04605 16724065 PMC2430087

[32] Taylor PD, Day T, Wild G. Evolution of cooperation in a finite homogeneous graph. Nature. 2007; 447(7143):469–472. 10.1038/nature05784 17522682

[33] Lehmann L, Keller L, Sumpter DJT. The evolution of helping and harming on graphs: the return of inclusive fitness effect. Journal of Evolutionary Biology. 2007; 20(6):2284–2295. 10.1111/j.1420-9101.2007.01414.x 17956391

[34] Ohtsuki H, Pacheco JM, Nowak MA. Evolutionary graph theory: Breaking the symmetry between interaction and replacement. Journal of Theoretical Biology. 2007; 246(4):681–694. 10.1016/j.jtbi.2007.01.024 17350049 PMC2396517

[35] Ohtsuki H, Nowak MA. Direct reciprocity on graphs. Journal of Theoretical Biology. 2007; 247(3):462–470. 10.1016/j.jtbi.2007.03.018 17466339 PMC2376797

[36] Taylor PD, Day T, Wild G. From inclusive fitness to fixation probability in homogeneous structured populations. Journal of Theoretical Biology. 2007; 249(1):101–110. 10.1016/j.jtbi.2007.07.006 17727893

[37] Nathanson CG, Tarnita CE, Nowak MA. Calculating evolutionary dynamics in structured populations. Plos Computational Biology. 2009; 5(12):e1000615 10.1371/journal.pcbi.1000615 20019806 PMC2787627

[38] Fu F, Nowak MA. Global migration can lead to stronger spatial selection than local migration. Journal of Statistical Physics. 2013; 151(3–4):637–653. 10.1007/s10955-012-0631-6 23853390 PMC3706309

[39] Fu F, Tarnita CE, Christakis NA, Wang L, Rand DG, Nowak MA. Evolution of in-group favoritism. Scientific Reports. 2012; 2:460 10.1038/srep00460 22724059 PMC3380441

[40] Zhang YL, Fu F, Chen XJ, Xie GM, Wang L. Cooperation in group-structured populations with two layers of interactions. Scientific Reports. 2015; 5:17446 10.1038/srep17446 26632251 PMC4668372

[41] Guo J, Zhao YL, Sun CY, Yu Y. Recursive identification of FIR systems with binary-valued outputs and communication channels. Automatica. 2015; 60:165–172. 10.1016/j.automatica.2015.06.030

[42] Guo J, Zhao YL, Sun CY. State estimation with quantised innovations and communication channels. IET Control Theory and Applications. 2015; 9(17):2606–2612. 10.1049/iet-cta.2015.0029

[43] He W, Ge SS. Vibration control of a flexible beam with output constraint. IEEE Transactions on Industrial Electronics. 2015; 62(8):5023–5030. 10.1109/TIE.2015.2400427

[44] He W, Zhang S, Ge SS. Adaptive control of a flexible crane system with the boundary output constraint. IEEE Transactions on Industrial Electronics. 2014; 61(8):4126–4133. 10.1109/TIE.2013.2288200

[45] Sun GQ, Chakrabort A, Liu QX, Jin Z, Anderson KE, Li BL. Influence of time delay and nonlinear diffusion on herbivore Outbreak. Communications in Nonlinear Science and Numerical Simulation. 2014; 19:1507–1518. 10.1016/j.cnsns.2013.09.016

[46] Sun GQ, Wang SL, Ren Q, Jin Z, Wu YP. Effects of time delay and space on herbivore dynamics: linking inducible defenses of plants to herbivore outbreak. Scientific Reports. 2015; 5:11246 10.1038/srep11246 26084812 PMC4471659

[47] Li L, Jin Z, Li J. Periodic solutions in a herbivore-plant system with time delay and spatial diffusion. Applied Mathematical Modelling. 2016; 40:4765–4777. 10.1016/j.apm.2015.12.003

[48] Strobeck C. Average number of nucleotide differences in a sample from a single subpopulation: a test for population subdivision. Genetics. 1987; 117(1):149–153. 17246396 10.1093/genetics/117.1.149 PMC1203183

