# Supplementary material for: Intermediate-Range Migration Furnishes a Narrow Margin of Efficiency in the Two-Strategy Competition
Source: PLoS One. 2016 May 24;11(5):e0155787. doi: 10.1371/journal.pone.0155787 (PMC4878735; doi:10.1371/journal.pone.0155787)
Supplement: S1 Text — (PDF) [file pone.0155787.s001.pdf]

S1 Text: Intermediate-range migration furnishes a narrow margin of efficiency in the two-strategy competition

Yanling Zhang[1], Qi Su[2], Changyin Sun[1]

[1] School of Automation and Electrical Engineering, University of Science and Technology Beijing, Beijing 100083, China

[2] Center for Systems and Control, State Key Laboratory for Turbulence and Complex Systems, College of Engineering, Peking University, Beijing, China

Here, we will show how $\langle\sum_{l,i,j} h_i\cdot h_j(1-s_l)s_is_j\rangle_0$, $\langle\sum_{l,i}(1-s_l)s_i\rangle_0$, and $\langle\sum_{l,i,j} h_i\cdot h_j(1-s_l)s_i(1-s_j)\rangle_0$ are expressed by the probabilities assigned to the events that three randomly chosen individuals have the given strategies and locations under neutral selection.

Let the expectation E be taken over all possible triples of (l, i, j) or all possible pairs of (l, i),

$$\begin{aligned}
&\langle\textstyle\sum_{l,i,j} h_i\cdot h_j(1-s_l)s_is_j\rangle_0 = N^3E[\langle h_i\cdot h_j(1-s_l)s_is_j\rangle_0],\\
&\langle\textstyle\sum_{l,i}(1-s_l)s_i\rangle_0 = N^2E[\langle(1-s_l)s_i\rangle_0],\\
&\langle\textstyle\sum_{l,i,j} h_i\cdot h_j(1-s_l)s_i(1-s_j)\rangle_0 = N^3E[\langle h_i\cdot h_j(1-s_l)s_i(1-s_j)\rangle_0].
\end{aligned}\tag{1}$$

The sum over l, i, j without limitation means that three individuals are chosen randomly and with replacement from the population: with probability $1/N^2$, all three individuals are identical ($l = i = j$); with probability $(N-1)/N^2$, two given individuals are the same but the third is different ($l = i \neq j$, $l = j \neq i$, or $i = j \neq l$); with probability $(N-1)(N-2)/N^2$, the three are different from each other ($l \neq i \neq j \neq l$). The sum over l, i without limitation means that two individuals are chosen randomly and with replacement from the population: with probability $1/N$, the two individuals are identical ($l = i$); with probability $(N-1)/N$, they are different ($l \neq i$). Therefore,

$$\begin{aligned}
E[\langle h_i\cdot h_j(1-s_l)s_is_j\rangle_0] &= \tfrac{N-1}{N^2}\langle s_i(1-s_j)|i\neq j\rangle_0 + \tfrac{(N-1)(N-2)}{N^2}\langle h_i\cdot h_j(1-s_l)\\
&\quad s_is_j|l\neq i\neq j\neq l\rangle_0,\\
E[\langle(1-s_l)s_i\rangle_0] &= \tfrac{N-1}{N}\langle(1-s_l)s_i|l\neq i\rangle_0,\\
E[\langle h_i\cdot h_j(1-s_l)s_i(1-s_j)\rangle_0] &= \tfrac{N-1}{N^2}\langle h_i\cdot h_js_i(1-s_j)|i\neq j\rangle_0 + \tfrac{(N-1)(N-2)}{N^2}\\
&\quad \langle h_i\cdot h_j(1-s_l)s_i(1-s_j)|l\neq i\neq j\neq l\rangle_0.
\end{aligned}\tag{2}$$

Each term of the right side can be understand as the probabilities assigned to the events that three randomly chosen individuals have the given strategies and locations under neutral selection.

Take $\langle h_i \cdot h_j(1-s_l)s_i s_j | l \neq i \neq j \neq l\rangle_0$ for example,

$$\begin{aligned}&\langle h_i \cdot h_j(1-s_l)s_i s_j | l \neq i \neq j \neq l\rangle_0 = \langle 1(h_i \cdot h_j = 1 - s_l = s_i = s_j = 1 \\ &| l \neq i \neq j \neq l)\rangle_0 = Pr(h_i \cdot h_j = 1, s_l = 0, s_i = 1, s_j = 1 | l \neq i \neq j \neq l),\end{aligned} \tag{3}$$

where the indicator function $1(X)$ is 1 if the event X is true and 0 otherwise, and $Pr(X)$ is the probability that X occurs. The two equal signs are self-evident. $Pr(h_i \cdot h_j = 1, s_l = 0, s_i = 1, s_j = 1 | l \neq i \neq j \neq l)$ can be understood as the probability that three randomly chosen individuals labelled by $1, 2, 3$ satisfy $h_2 \cdot h_3 = 1$, $s_1 = 0$, $s_2 = 1$, $s_3 = 1$. Let $Pr(s_1 = a, s_2 = b, s_3 = c, h_2 \cdot h_3 = 1) = Pr(h_i \cdot h_j = 1, s_l = a, s_i = b, s_j = c \, | l \neq i \neq j \neq l)$ for simplicity,

$$\langle h_i \cdot h_j(1-s_l)s_i s_j | l \neq i \neq j \neq l\rangle_0 = Pr(s_1 = 0, s_2 = 1, s_3 = 1, h_2 \cdot h_3 = 1). \tag{4}$$

Similar to Eq. (4), other terms of the right side in Eq. (2) are given by

$$\begin{aligned}&\langle s_i(1-s_j) | i \neq j\rangle_0 = Pr(s_1 = 1, s_2 = 0), \\ &\langle h_i \cdot h_j s_i(1-s_j) | i \neq j\rangle_0 = Pr(s_1 = 1, s_2 = 0, h_1 \cdot h_2 = 1), \\ &\langle h_i \cdot h_j(1-s_l)s_i(1-s_j) | l \neq i \neq j \neq l\rangle_0 = Pr(s_1 = 0, s_2 = 1, s_3 = 0, h_2 \cdot h_3 = 1),\end{aligned} \tag{5}$$

where $Pr(s_1 = a, s_2 = b) = Pr(s_i = a, s_j = b | i \neq j)$ ($Pr(s_1 = a, s_2 = b, h_1 \cdot h_2 = 1) = Pr(s_i = a, s_j = b, h_i \cdot h_j = 1 | i \neq j)$) is the probability that two randomly chosen individuals labelled by $1, 2$ satisfy $s_1 = a$, $s_2 = b$ ($s_1 = a, s_2 = b, h_1 \cdot h_2 = 1$).

Eqs. (1), (2), (4), and (5) yield

$$\begin{aligned}&\langle \textstyle\sum_{l,i,j} h_i \cdot h_j(1-s_l)s_i s_j\rangle_0 = N(N-1)Pr(s_1 = 1, s_2 = 0) + N(N-1)(N-2) \\ &\qquad Pr(s_1 = 0, s_2 = 1, s_3 = 1, h_2 \cdot h_3 = 1), \\ &\langle \textstyle\sum_{l,i}(1-s_l)s_i\rangle_0 = N(N-1)Pr(s_1 = 1, s_2 = 0), \\ &\langle \textstyle\sum_{l,i,j} h_i \cdot h_j(1-s_l)s_i(1-s_j)\rangle_0 = N(N-1)Pr(s_1 = 1, s_2 = 0, h_1 \cdot h_2 = 1)+ \\ &\qquad N(N-1)(N-2)Pr(s_1 = 0, s_2 = 1, s_3 = 0, h_2 \cdot h_3 = 1).\end{aligned} \tag{6}$$
