# Supplementary material for: Intermediate-Range Migration Furnishes a Narrow Margin of Efficiency in the Two-Strategy Competition
Source: PLoS One. 2016 May 24;11(5):e0155787. doi: 10.1371/journal.pone.0155787 (PMC4878735; doi:10.1371/journal.pone.0155787)
Supplement: S2 Text — (PDF) [file pone.0155787.s002.pdf]

S2 Text: Intermediate-range migration furnishes a narrow margin of efficiency in the two-strategy competition

Yanling Zhang[1], Qi Su[2], Changyin Sun[1]

[1] School of Automation and Electrical Engineering, University of Science and Technology Beijing, Beijing 100083, China

[2] Center for Systems and Control, State Key Laboratory for Turbulence and Complex Systems, College of Engineering, Peking University, Beijing, China

Here, we will calculate the concrete expression of the probability that three different individuals labelled $1,2,3$ satisfy $s_1=\delta_1, s_2=\delta_2, s_3=\delta_3, h_2\cdot h_3=1$.

When the optional strategies for an individual are $\{1,2,\cdots,S\}$, the general expression of $Pr(s_1=\delta_1, s_2=\delta_2, s_3=\delta_3, h_2\cdot h_3=1)$ has been given by

$$\begin{aligned}&Pr(s_1=\delta_1, s_2=\delta_2, s_3=\delta_3, h_2\cdot h_3=1)=\frac{1}{3MS^3}\sum_{z_1=M,z_2+z_3=M or 2M}\sum_{w_1+w_2+w_3=S,2S or 3S}\{\sum_{x_1=z_1+z_2,x_2=z_3,y_1=w_1+w_2,y_2=w_3}+\sum_{x_1=z_1+z_3,x_2=z_2,y_1=w_1+w_3,y_2=w_2}\\&+\sum_{x_1=z_2+z_3,x_2=z_1,y_1=w_2+w_3,y_2=w_1}\}\Phi(f(z_1),f(z_2),f(z_3),g(w_1),g(w_2),g(w_3))\Psi(f(x_1),f(x_2),g(y_1),g(y_2))\exp(-\frac{2\pi i}{S}(w_1\cdot\delta_1+w_2\cdot\delta_2+w_3\cdot\delta_3)),\end{aligned}\tag{1}$$

where

$$\Psi(f(x_1),f(x_2),g(y_1),g(y_2))=\frac{(1-u)(1-v)+(1-u)v\sum_{i=1}^2 f(x_i)/2+(1-v)u\sum_{i=1}^2 g(y_i)/2+uv\sum_{i=1}^2 f(x_i)g(y_i)/2}{1+(N-1)(1-u)v(1-\sum_{i=1}^2\frac{f(x_i)}{2})+(N-1)(1-v)u(1-\sum_{i=1}^2\frac{g(y_i)}{2})+(N-1)uv(1-\sum_{i=1}^2\frac{f(x_i)g(y_i)}{2})},\tag{2}$$

$$\Phi(f(z_1),f(z_2),f(z_3),g(w_1),g(w_2),g(w_3))=\begin{cases}\frac{2(1-u)(1-v)+(1-u)v(f(z_1)+f(z_2))+(1-v)u(g(w_1)+g(w_2))+uv(f(z_1)g(w_1)+f(z_2)g(w_2))}{2+(N-2)(1-u)v(1-\sum_{i=1}^3\frac{f(z_i)}{3})+(N-2)(1-v)u(1-\sum_{i=1}^3\frac{g(w_i)}{3})+(N-2)uv(1-\sum_{i=1}^3\frac{f(z_i)g(w_i)}{3})},\\ \quad\text{if } x_1=z_1+z_2, x_2=z_3, y_1=w_1+w_2, y_2=w_3;\\ \frac{2(1-u)(1-v)+(1-u)v(f(z_1)+f(z_3))+(1-v)u(g(w_1)+g(w_3))+uv(f(z_1)g(w_1)+f(z_3)g(w_3))}{2+(N-2)(1-u)v(1-\sum_{i=1}^3\frac{f(z_i)}{3})+(N-2)(1-v)u(1-\sum_{i=1}^3\frac{g(w_i)}{3})+(N-2)uv(1-\sum_{i=1}^3\frac{f(z_i)g(w_i)}{3})},\\ \quad\text{if } x_1=z_1+z_3, x_2=z_2, y_1=w_1+w_3, y_2=w_2;\\ \frac{2(1-u)(1-v)+(1-u)v(f(z_2)+f(z_3))+(1-v)u(g(w_2)+g(w_3))+uv(f(z_2)g(w_2)+f(z_3)g(w_3))}{2+(N-2)(1-u)v(1-\sum_{i=1}^3\frac{f(z_i)}{3})+(N-2)(1-v)u(1-\sum_{i=1}^3\frac{g(w_i)}{3})+(N-2)uv(1-\sum_{i=1}^3\frac{f(z_i)g(w_i)}{3})},\\ \quad\text{if } x_1=z_2+z_3, x_2=z_1, y_1=w_2+w_3, y_2=w_1.\end{cases}\tag{3}$$

$g(x)$ $(f(x))$ corresponds to the structure function of the random walk describing the mutation process (the migration process) along a lineage and satisfies $g(S)=1$ and $g(x)=g(S-x)$ $(f(M)=1$

and $f(x) = f(M - x)$). It is noteworthy that w_1, w_2, w_3, y_1, y_2 (z_1, z_2, z_3, x_1, x_2) can take on only integers between 1 and S (M) including the boundaries.

In our model ($S = 2$), the set $\{w_1 + w_2 + w_3 = 2, 4\, or\, 6\}$ in Eq. (1) includes four points $B_1 = \{w_1 = w_2 = w_3 = 2\}$, $B_2 = \{w_1 = 2, w_2 = w_3 = 1\}$, $B_3 = \{w_2 = 2, w_1 = w_3 = 1\}$, and $B_4 = \{w_3 = 2, w_1 = w_2 = 1\}$. The offspring will adopt one of the two optional strategies once a mutation occurs, then $g(x) = \frac{1}{2} + \frac{1}{2}\cos(\pi x)$ and the values of $g(w_1)$, $g(w_2)$, $g(w_3)$ over these points are as follows:

$$\begin{cases} g(w_1) = 1, g(w_2) = 1, g(w_3) = 1, & \text{for } B_1; \\ g(w_1) = 1, g(w_2) = 0, g(w_3) = 0, & \text{for } B_2; \\ g(w_1) = 0, g(w_2) = 1, g(w_3) = 0, & \text{for } B_3; \\ g(w_1) = 0, g(w_2) = 0, g(w_3) = 1, & \text{for } B_4. \end{cases} \tag{4}$$

Meanwhile, the set $\{(z_1, z_2, z_3)|z_1 = M, z_2 + z_3 = M \text{ or } 2M\}$ in Eq. (1) satisfies

$$f(z_1) = 1, \quad f(z_2) = f(z_3). \tag{5}$$

In the case of $\{x_1 = z_1 + z_2, x_2 = z_3, y_1 = w_1 + w_2, y_2 = w_3\}$,

$$\begin{cases} g(y_1) = 1, \quad g(y_2) = 1, & \text{for } B_1 \text{ and } B_4; \\ g(y_1) = 0, \quad g(y_2) = 0, & \text{for } B_2 \text{ and } B_3; \\ f(x_1) = f(x_2) = f(z_2), & \text{for } z_1 = M, z_2 + z_3 = M \text{ or } 2M. \end{cases} \tag{6}$$

Substituting Eqs. (4) and (5) into Eq. (3) yields

$$\Phi(\cdots) = \begin{cases} L_1(f(z_2)) = \frac{2-v+vf(z_2)}{2+\frac{2(N-2)v}{3}(1-f(z_2))}, & \text{for } B_1; \\ L_2(f(z_2)) = \frac{1+(1-u)(1-v+vf(z_2))}{2+\frac{2(N-2)u}{3}+\frac{2(N-2)(1-u)v}{3}(1-f(z_2))}, & \text{for } B_2; \\ \Phi_1(f(z_2)) = \frac{2-u-v+vf(z_2)}{2+\frac{2(N-2)u}{3}+\frac{(N-2)(2-u)v}{3}(1-f(z_2))}, & \text{for } B_3; \\ \Phi_2(f(z_2)) = \frac{(1-u)(2-v+vf(z_2))}{2+\frac{2(N-2)u}{3}+\frac{(N-2)(2-u)v}{3}(1-f(z_2))}, & \text{for } B_4. \end{cases} \tag{7}$$

Substituting Eq. (6) into Eq. (2) leads to

$$\Psi(\cdots) = \begin{cases} \Psi_1(f(z_2)), & \text{for } B_1 \text{ and } B_4; \\ \Psi_2(f(z_2)), & \text{for } B_2 \text{ and } B_3. \end{cases} \tag{8}$$

In the case of $\{x_1 = z_1 + z_3, x_2 = z_2, y_1 = w_1 + w_3, y_2 = w_2\}$,

$$\begin{cases} g(y_1) = 1, \quad g(y_2) = 1, & \text{for } B_1 \text{ and } B_3; \\ g(y_1) = 0, \quad g(y_2) = 0, & \text{for } B_2 \text{ and } B_4; \\ f(x_1) = f(x_2) = f(z_2), & \text{for } z_1 = M, z_2 + z_3 = M \text{ or } 2M. \end{cases} \tag{9}$$

Substituting Eqs. (4) and (5) into Eq. (3) yields

$$\Phi(\cdots) = \begin{cases} L_1(f(z_2)), & \text{for } B_1; \\ L_2(f(z_2)), & \text{for } B_2; \\ \Phi_2(f(z_2)), & \text{for } B_3; \\ \Phi_1(f(z_2)), & \text{for } B_4. \end{cases} \tag{10}$$

Substituting Eq. (9) into Eq. (2) leads to

$$\Psi(\cdots) = \begin{cases} \Psi_1(f(z_2)), & \text{for } B_1 \text{ and } B_3; \\ \Psi_2(f(z_2)), & \text{for } B_2 \text{ and } B_4. \end{cases} \tag{11}$$

In the case of $\{x_1 = z_2 + z_3, x_2 = z_1, y_1 = w_2 + w_3, y_2 = w_1\}$,

$$\begin{cases} g(y_1) = 1, \quad g(y_2) = 1, & \text{for } B_1 \text{ and } B_2; \\ g(y_1) = 0, \quad g(y_2) = 0, & \text{for } B_3 \text{ and } B_4; \\ f(x_1) = 1, \quad f(x_2) = 1, & \text{for } z_1 = M, z_2 + z_3 = M \text{ or } 2M. \end{cases} \tag{12}$$

Substituting Eqs. (4) and (5) into Eq. (3) yields

$$\Phi(\cdots) = \begin{cases} L_3(f(z_2)) = \frac{1-v+vf}{1+\frac{(N-2)v}{3}(1-f(z_2))}, & \text{for } B_1; \\ L_4(f(z_2)) = \frac{(1-u)(1-v+vf(z_2))}{1+\frac{(N-2)u}{3}+\frac{(N-2)(1-u)v}{3}(1-f(z_2))}, & \text{for } B_2; \\ \Phi_3(f(z_2)) = \frac{(2-u)(1-v+vf(z_2))}{2+\frac{2(N-2)u}{3}+\frac{(N-2)(2-u)v}{3}(1-f(z_2))}, & \text{for } B_3; \\ \Phi_3(f(z_2)), & \text{for } B_4. \end{cases} \tag{13}$$

Substituting Eq. (12) into Eq. (2) leads to

$$\Psi(\cdots) = \begin{cases} 1, & \text{for } B_1 \text{ and } B_2; \\ \alpha_1 = \frac{1-u}{1+(N-1)u}, & \text{for } B_3 \text{ and } B_4. \end{cases} \tag{14}$$

TABLE I: **The expressions of $\Phi(f(z_1), f(z_2), f(z_3), g(w_1), g(w_2), g(w_3))\Psi(f(x_1), f(x_2), g(y_1), g(y_2))$ for different points B_1, B_2, B_3, B_4 and different coalescence combinations.**

	$x_1 = z_1 + z_2, x_2 = z_3$ $y_1 = w_1 + w_2, y_2 = w_3$	$x_1 = z_1 + z_3, x_2 = z_2$ $y_1 = w_1 + w_3, y_2 = w_2$	$x_1 = z_2 + z_3, x_2 = z_1$ $y_1 = w_2 + w_3, y_2 = w_1$
B_1	$L_1(f(z_2))\Psi_1(f(z_2))$	$L_1(f(z_2))\Psi_1(f(z_2))$	$L_3(f(z_2))$
B_2	$L_2(f(z_2))\Psi_2(f(z_2))$	$L_2(f(z_2))\Psi_2(f(z_2))$	$L_4(f(z_2))$
B_3	$\Phi_1(f(z_2))\Psi_2(f(z_2))$	$\Phi_2(f(z_2))\Psi_1(f(z_2))$	$\Phi_3(f(z_2))\alpha_1$
B_4	$\Phi_2(f(z_2))\Psi_1(f(z_2))$	$\Phi_1(f(z_2))\Psi_2(f(z_2))$	$\Phi_3(f(z_2))\alpha_1$

According to Eqs. (7), (8), (10), (11), (13), and (14), the expressions of $\Phi(f(z_1), f(z_2), f(z_3), g(w_1), g(w_2), g(w_3))\Psi(f(x_1), f(x_2), g(y_1), g(y_2))$ for different points B_1, B_2, B_3, B_4 and different coalescence combinations are summarized in Table I.

The values of $\exp\{-\pi i(\sum_{x=1}^{3} \delta_x w_x)\}$ for B_1, B_2, B_3, B_4 and different strategy combinations of three individuals are summarized in Table II.

TABLE II: **The values of $\exp\{-\pi i(\sum_x^3 \delta_x w_x)\}$ for B_1, B_2, B_3, B_4 and different strategy combinations of three individuals**

	$\delta_1 = \delta_2 = \delta_3$	$\delta_1 = \delta_2 \neq \delta_3$	$\delta_1 = \delta_3 \neq \delta_2$	$\delta_2 = \delta_3 \neq \delta_1$
B_1	1	1	1	1
B_2	1	−1	−1	1
B_3	1	−1	1	−1
B_4	1	1	−1	−1

By substituting Table I and Table II into Eq. (1) and using $L_3(f) + 2L_1(f)\Psi_1(f) = 3\Psi_1(f)$,

$L_4(f) + 2L_2(f)\Psi_2(f) = 3\Psi_2(f)$, we have

$$P(s_1 = \delta_1, s_2 = \delta_2, s_3 = \delta_3, h_2 \cdot h_3 = 1) = \frac{1}{24M}\sum_{x=1}^{M}$$

$$\begin{cases} 3\Psi_1(f(x)) + 3\Psi_2(f(x)) + 2(\Phi_1(f(x))\Psi_2(f(x)) + \Phi_2(f(x))\Psi_1(f(x))+ \\ \qquad \Phi_3(f(x))\alpha_1), & \text{if } \delta_1 = \delta_2 = \delta_3; \\ 3\Psi_1(f(x)) - 3\Psi_2(f(x)), & \text{if } \delta_1 = \delta_2 \neq \delta_3; \\ 3\Psi_1(f(x)) - 3\Psi_2(f(x)), & \text{if } \delta_1 = \delta_3 \neq \delta_2; \\ 3\Psi_1(f(x)) + 3\Psi_2(f(x)) - 2(\Phi_1(f(x))\Psi_2(f(x)) + \Phi_2(f(x))\Psi_1(f(x))+ \\ \Phi_3(f(x))\alpha_1), & \text{if } \delta_2 = \delta_3 \neq \delta_1; \end{cases} \tag{15}$$

where

$\Psi_1(f) = \frac{1-v+vf}{1+(N-1)v(1-f)}$, $\Psi_2(f) = \frac{(1-u)(1-v+vf)}{1+(N-1)u+(N-1)(1-u)v(1-f)}$,

$\Phi_1(f) = \frac{2-u-v+vf}{2+\frac{2(N-2)u}{3}+\frac{(N-2)(2-u)v}{3}(1-f)}$, $\Phi_2(f) = \frac{(1-u)(2-v+vf)}{2+\frac{2(N-2)u}{3}+\frac{(N-2)(2-u)v}{3}(1-f)}$,

$\Phi_3(f) = \frac{(2-u)(1-v+vf)}{2+\frac{2(N-2)u}{3}+\frac{(N-2)(2-u)v}{3}(1-f)}$, $\alpha_1 = \frac{1-u}{1+(N-1)u}$.
