# Supplementary material for: Intermediate-Range Migration Furnishes a Narrow Margin of Efficiency in the Two-Strategy Competition
Source: PLoS One. 2016 May 24;11(5):e0155787. doi: 10.1371/journal.pone.0155787 (PMC4878735; doi:10.1371/journal.pone.0155787)
Supplement: S3 Text — (PDF) [file pone.0155787.s003.pdf]

S3 Text: Intermediate-range migration furnishes a narrow margin of efficiency in the two-strategy competition

Yanling Zhang[1], Qi Su[2], Changyin Sun[1]

[1] School of Automation and Electrical Engineering, University of Science and Technology Beijing, Beijing 100083, China

[2] Center for Systems and Control, State Key Laboratory for Turbulence and Complex Systems, College of Engineering, Peking University, Beijing, China

The general expression of the probability that two different individuals labelled 1, 2 satisfy $s_1 = \delta_1$, $s_2 = \delta_2$, $m_1 = \gamma_1$, $m_2 = \gamma_2$ when the optional strategies for an individual are $\{1, 2, \cdots, S\}$ has been given

$$
\begin{aligned}
&Pr(s_1 = \delta_1, s_2 = \delta_2, m_1 = \gamma_1, m_2 = \gamma_2) \\
&= \tfrac{1}{M^2 S^2} \textstyle\sum_{x_1+x_2=M \,\mathrm{or}\, 2M} \sum_{y_1+y_2=S \,\mathrm{or}\, 2S} \Psi(f(x_1), f(x_2), g(y_1), g(y_2)) \exp(-\tfrac{2\pi i}{M} \\
&(x_1 \cdot \gamma_1 + x_2 \cdot \gamma_2)) \exp(-\tfrac{2\pi i}{S}(y_1 \cdot \delta_1 + y_2 \cdot \delta_2)),
\end{aligned} \tag{1}
$$

In our model ($S = 2$), the offspring will adopt one of the two optional strategies equi-probably once the mutation occurs, then $g(x) = \frac{1}{2} + \frac{1}{2}\cos(\pi x)$ and we have

$$
\begin{aligned}
&Pr(s_1 = \delta_1, s_2 = \delta_2) = \textstyle\sum_{\gamma_1,\gamma_2} P(s_1 = \delta_1, s_2 = \delta_2; m_1 = \gamma_1, m_2 = \gamma_2) \\
&= \tfrac{1}{S^2} \textstyle\sum_{y_1+y_2=S \,\mathrm{or}\, 2S} \Psi(f(M), f(M), g(y_1), g(y_2)) \exp(-\tfrac{2\pi i}{S}(y_1 \cdot \delta_1 + y_2 \cdot \delta_2)) \\
&= \tfrac{1}{4}\Psi(f(M), f(M), g(1), g(1)) \exp(\pi i(\delta_1 + \delta_2)) + \tfrac{1}{4}\Psi(f(M), f(M), g(2), g(2)),
\end{aligned} \tag{2}
$$

where $\Psi(f(M), f(M), g(1), g(1)) = \frac{1-u}{1+(N-1)u} = \alpha_1$ and $\Psi(f(M), f(M), g(2), g(2)) = 1$. Therefore,

$$
Pr(s_1 = \delta_1, s_2 = \delta_2) = \begin{cases} \frac{1}{4}(1 + \alpha_1), & \text{if } \delta_1 = \delta_2; \\ \frac{1}{4}(1 - \alpha_1), & \text{if } \delta_1 \neq \delta_2. \end{cases} \tag{3}
$$
